# Estrous Cycle and Gestational Age-Dependent Expression of Members of the Interleukin-36 Subfamily in a Semi-Allogeneic Model of Infected and Non-Infected Murine Pregnancy

**DOI:** 10.3389/fimmu.2016.00376

**Published:** 2016-09-22

**Authors:** José Martin Murrieta-Coxca, Fernando Gómez-Chávez, Damariz Adriana Baeza-Martínez, Mario Eugenio Cancino-Diaz, Juan Carlos Cancino-Diaz, Sonia Mayra Pérez-Tapia, Elba Reyes-Maldonado, Sandra Rodríguez-Martínez

**Affiliations:** ^1^Departamento de Inmunología, Instituto Politécnico Nacional, Escuela Nacional de Ciencias Biológicas, Mexico City, Mexico; ^2^Instituto Nacional de Pediatría, SSA, Laboratorio de Inmunología Experimental, Cátedra CONACyT, Mexico City, Mexico; ^3^Departamento de Microbiología, Instituto Politécnico Nacional, Escuela Nacional de Ciencias Biológicas, Mexico City, Mexico; ^4^Facultad de Estudios Superiores-Iztacala, UNAM, Los Reyes Iztacala Tlalnepantla, Mexico City, Mexico; ^5^Departamento de Morfología, Instituto Politécnico Nacional, Escuela Nacional de Ciencias Biológicas, Mexico City, Mexico

**Keywords:** IL-36, estrous cycle, pregnancy, *Listeria monocytogenes*, inflammation, mRNA, protein, epithelium

## Abstract

The IL-36 subfamily is a recently described group of cytokines with pro-inflammatory behavior, comprising three agonists (α, β, and γ), its receptor (R), and one antagonist (Ra). The expression and function of IL-36 subfamily members in the estrous cycle in healthy and infected pregnancy has not been described. We evaluated mRNA and protein expression of IL-36 family members during the estrous cycle, implantation, fetal development, and post-labor periods in a model of allogenic pregnancy in mice. We also explored the ability of *Listeria monocytogenes* to modulate the expression of IL-36 subfamily members during pregnancy. Expression of IL-36 subfamily members showed different expression during the estrous cycle and pregnancy but was induced at estrous, 16.5 days *post coitum* (dpc), 18.5 dpc, and labor. IL-36 subfamily members showed a characteristic distribution in the glandular epithelium, perimetrium, myometrium, and stratum vasculare. Infection with *L. monocytogenes* during pregnancy induced strong production of IL-36 subfamily members, an observation that correlated with an increasing prevalence of fetal loss. In conclusion, IL-36 agonists showed specific patterns of mRNA and protein expression that might suggest functional specialization or specific target cells. Infection with *L. monocytogenes* during pregnancy induced strong production of IL-36 subfamily members.

## Introduction

The menstrual cycle in humans, as well as the estrous cycle in rodents, prepares females for reproduction (1). Establishment of pregnancy requires a finely balanced interaction between the embryo and maternal tissue that is mediated by hormones and cytokines (2). Several studies in mammals have shown that inflammation is crucial at the maternal–fetal interface, both at implantation and at parturition (3, 4). During implantation and placentation, the uterus must be modified to accept the graft. Such tissue remodeling is orchestrated by the activity of several cytokines and chemokines arising from uterine natural killer cells, macrophages, dendritic cells (DCs), and non-immune uterine cells (4). In health, labor is predicated to be an inflammatory event because of increased leukocyte infiltrates and the production of pro-inflammatory molecules in the uterus (5). Leukemia inhibitory factor (LIF), IL-6, TNF-α, and IL-1β have been described as the main participants in inflammation during the events cited above. Other molecules of the IL-1 family (IL-1α, IL-18, IL-1R1, IL-1RA, IL-1RAcP, and ST2L) and molecules associated with inflammasomes (NLRP-1, NLRP-3, NLRC-4, caspase I, and ASC) have been reported to be active in early pregnancy, parturition, preterm labor, preeclampsia, and infection during pregnancy (3, 4, 6).

Members of the IL-1 subfamily, such as IL-36α (IL-1F6), IL-36β (IL-1F8), IL-36γ (IL-1F9), IL-36R (IL-1Rrp2), and the receptor antagonist IL-36Ra (IL-1F5) constitute a novel signaling system that has not been studied in depth in the normal inflammatory process of pregnancy, or in pregnant women with a bacterial infection.

There is evidence of IL-36 involvement in the innate and adaptive immunity associated with inflammation enhancement in the skin, kidney, joints, brain, and lung, where keratinocytes, CD4^+^ T cells, DCs, and macrophages can be a source of members of the IL-36 subfamily (7). However, the extent of expression of IL-36 in healthy or infected uteri remains unknown. Most studies on members of the IL-36 family have focused on chronic inflammatory disorders, such as general pustular psoriasis, rheumatoid arthritis, tubulointerstitial lesions, inflammation of brain tissue, airway inflammation, and gingivitis (7). IL-36α, β, and γ are expressed by several cell types, but epithelial cells (keratinocytes) are potent producers of these cytokines and, importantly, they are overexpressed in psoriatic skin lesions (8). Transgenic mice overexpressing IL-36 in skin show phenotypic lesions related to those observed in human psoriasis, which suggests a pro-inflammatory role of these cytokines (9). Immune cells, such as DCs, macrophages, T helper (Th) cells, and granulocytes, are also important producers and responders to IL-36, and they also participate in pro-inflammatory diseases, such as psoriasis (10).

We explored the expression of IL-36 subfamily members in the uteri of pregnant mice infected and not infected by *Listeria monocytogenes*. Our results showed that these cytokines show differential upregulation of expression during estrus, implantation, and labor, suggesting that members of the IL-36 subfamily have different functions in their pro-inflammatory roles. Our results contribute to understanding of the immunologic processes activated during reproduction.

## Materials and Methods

### Ethical Approval of the Study Protocol

Experiments were carried out according to the relevant guidelines for animal use as approved by the Institutional Bioethics Committee of Escuela Nacional de Ciencias Biológicas-IPN (Ciudad de México, Mexico). These guidelines are based on the *Guide for the Care and Use of Laboratory Animals* (National Institutes of Health, Bethesda, MD, USA).

### Mating between Non-Infected Mice

Healthy, virgin, and sexually mature ICR female mice (8 weeks) were obtained from the Animal Center Facilities of Escuela Superior de Medicina-IPN (Mexico City, Mexico). Mice were housed in a room at constant temperature (22°C) with a fixed 12-h light–dark cycle and had access to food and water *ad libitum*.

Stage of the estrous cycle was determined by cytologic evaluation of vaginal smears. From mice with two continuous estrous cycles, uteri were collected at diestrus, proestrus, estrus, and metestrus (*n* = 4 mice per stage).

Healthy female mice at the estrus stage were mated with healthy C57BL/6 male mice overnight. The following morning, spermatozoids in females were verified with a vaginal smear and, if spermatozoids were present, the mice were considered to be 0.5 days *post coitum* (dpc).

Four uteri from pregnant mice were collected 4.5, 5.5, 7.5, and 10.5 dpc (“peri-implantation” period); 12.5, 16.5, 18.5 dpc (“fetal development”); 19.5 dpc (“labor”); 2 days post-labor; 5 days post-labor. Uteri were extracted to measure the levels of mRNA and protein for IL-36 subfamily members. Samples were also embedded in paraffin for immunofluorescent analyses.

### Infection of BALB/c Mice with *L. monocytogenes*

A suspension of infective *L. monocytogenes* was prepared in sterile phosphate-buffered saline (PBS) at 10^9^ CFU/mL. Female BALB/c mice were inoculated (i.v.) in the tail with 100 μL of the *L. monocytogenes* suspension (10^8^ CFU/mouse) during estrus.

After infection, mice showed typical piloerection after day 3. Then, mice were again cycled and mated overnight with a healthy C57BL/6 male to obtain semiallogenic offspring. A procedure identical to that described above was carried out for non-infected mice to determine pregnancy. Implantation sites from pregnant non-infected and infected BALB/c mice were obtained at diestrus and at estrus, as well as at 4.5, 5.5, 7.5, and 10.5 dpc. To confirm infection with *L. monocytogenes* in harvested uteri, CFU/mL was determined, and a polymerase chain reaction (PCR) coupled to the DNA sequence of the 16S ribosome was carried out (data not shown).

### Semi-Quantitative Reverse Transcription-Polymerase Chain Reaction

Uteri were homogenized individually, and RNA was isolated using TRIzol^®^ reagent (Invitrogen, Carlsbad, CA, USA). Total RNA concentration was evaluated using a NanoDrop spectrophotometer (Thermo Scientific, Wilmington, DE, USA). Total RNA (2 μg) was reacted with DNAse I (Affymetrix, Santa Clara, CA, USA). cDNA was synthesized using MLV reverse transcriptase (Invitrogen). PCR reactions (20 μL) were carried out using 1 μL of the cDNA reaction, 10 μL of AmpliTaq Gold^®^ Fast PCR Master Mix (Life Technologies, Gaithersburg, MD, USA), and 0.2 μM-each of IL-36R, IL-36α, IL-36β, IL-36γ, and IL-36Ra primers or the glyceraldehyde 3-phosphate dehydrogenase (GAPDH) primer (housekeeping gene) (Table 1). Optimized PCR conditions were 35 cycles at 96°C for 5 s, 60°C for 5 s, and 68°C for 5 s. Amplified DNA (250 bp) was analyzed on a 2% agarose gel and stained using RedGel (Biotium Inc., CA, USA). Gel images were acquired in a ChemiDoc-It™ transilluminator (UVP, Upland, CA, USA), and the integrated pixel density (PD) of each band was calculated using AlphaImager^®^ (ProteinSimple, San Jose, CA, USA). The PD of each gene band was normalized by dividing the PD of the sample by that of the corresponding housekeeping gene (GAPDH) band. The change in the expression of each gene was calculated by dividing the expression of the normalized gene at a specified point in pregnancy by that of the expression of the normalized gene at diestrus.

**Table 1:** **Sequences of oligonucleotides**.

Gene/mouse	Sequence
GAPDH	FWD: 5′-CTACCCCCAATGTGTCCGTC-3′
	REV: 5′-GCCGTATTCATTGTCATACCAGG-3′
IL-36α	FWD: 5′-CATGGATCCTCACAATCTCCCA-3′
	REV: 5′-ACTTCCTTAAGCGCAAAGTTGG-3′
IL-36β	FWD: 5′-CATGGATCCTCACAATCTCCCA-3′
	REV: 5′-GCGCAAAGTTGGTTTGCCC-3′
IL-36γ	FWD: 5′-ACACCCATTTTCTACACACATCT-3′
	REV: 5′-AGCAGCAAAGTAGGGTGTCC-3′
IL-36R	FWD: 5′-GCAGCAGATACGTGTGAGGAC-3′
	REV: 5′-TTGGTAGCAGTTGTGGGCATT-3′
IL-36Ra	FWD: 5′-GGGCACTATGCTTCCGAATG-3′
	REV: 5′-CTTTGATTCCTGGCCCCCGA-3′

### Western Blotting

A total protein fraction (coming from 3 implantation sites or 1/2 uterus from non-pregnant mice) was extracted from uteri using 400 μL of RIPA buffer [Tris-HCl (pH 7.6), NaCl (150 mM), EDTA (2 mM), glycerol (10%), Triton-X100 (1%), sodium desoxicolate (0.5%), sodium lauryl sulfate (0.2%)], 1 mM of phenylmethylsulfonyl fluoride (Sigma–Aldrich, Saint Louis, MO, USA), and 1× complete protease inhibitor cocktail (Roche Diagnostics, Mannheim, Germany). Proteins were quantified using the Lowry method (Bio-Rad Laboratories, Hercules, CA, USA); 25 μg of protein extract were diluted 1:5 in Laemmli sample buffer (Bio-Rad Laboratories) containing 2-mercaptoethanol (Bio-Rad) and denatured by boiling. Samples were loaded on SDS–PAGE 15% gels and transferred to cellulose sheets (Amersham Biosciences, Amersham, UK). Non-specific binding was blocked with 4% gelatin in PBS 1× containing 0.5% Tween-20 for 1 h at 37°C. Specific primary antibodies to polyclonal goat anti-IL-36α, anti-IL-36β, anti-IL-36γ, anti-IL-36R, and anti-IL-36Ra (Santa Cruz Biotechnology, Santa Cruz, CA, USA) were diluted 1:750 in blocking buffer. The secondary antibody was horseradish peroxidase–IgG mouse anti-goat (1:5000 dilution). Chemiluminescence was developed using an electrochemiluminescence western blotting substrate (Pierce Biotechnology, Waltham, MA, USA) and detected on a ChemiDoc™ Touch imaging system (Bio-Rad).

### Immunofluorescence Assay

Uteri were fixed in 4% formalin and embedded in paraffin. Tissue slices (thickness, 6 μm) were obtained using a microtome (RM2125RT; Leica, Wetzlar, Germany) and placed on slides pre-coated with poly-l-lysine. Antigen retrieval was achieved by heating in a pressure cooker at 95°C for 15 min followed by immersion in antigen retrieval buffer (Life Technologies, Carlsbad, CA, USA). Samples were blocked with 0.1% albumin solution and incubated with primary antibody (anti-IL-36α, anti-IL-36β, anti-IL-36γ, anti-IL-36R, or anti-IL-36Ra) diluted in PBS for 50 min at 37°C. After two washes, the secondary antibody (rabbit fluorescein isothiocyanate–IgG anti-goat IgG) was added to samples at 1:1500 dilution and incubated for 1 h at 37°C. Preparations were observed under a confocal microscope (LSM 700; Zeiss, Oberkochen, Germany) for image capture. Captures were analyzed using LSM v5 Image Examiner software (Zeiss).

### Statistical Analyses

Results are the mean ± SEM. Statistical analyses were carried out using GraphPad Prism (GraphPad, San Diego, CA, USA). Differences between samples were evaluated using Newman–Keuls multiple comparison tests, after one-way ANOVA. *p* ≤ 0.05 was considered significant.

## Results

### Expression of IL-36 Subfamily Members in Non-Infected Uteri during the Estrous Cycle

**Figure 1:**
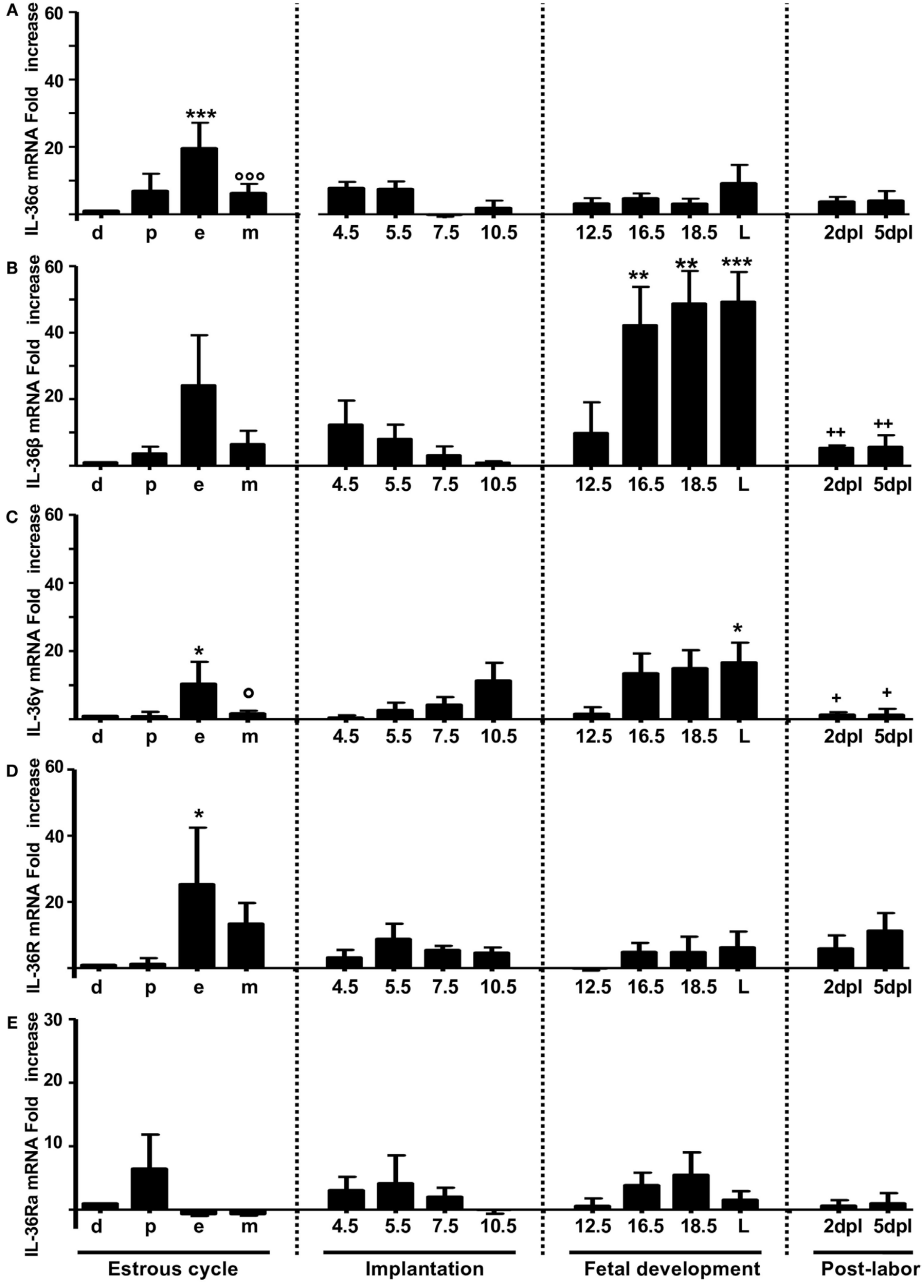
**mRNA expression of IL-36 family members in non-pregnant and pregnant mice**. IL-36α **(A)**, β **(B)**, γ **(C)**, IL-36R **(D)**, and IL-36Ra **(E)**. mRNA levels are expressed as fold increases normalized to GAPDH and relative to diestrus of non-infected mice. PCR results are shown as the mean ± SE. *p ≤ 0.05, **p ≤ 0.01, ***p ≤ 0.001 for significant difference between groups. d, diestrus; p, proestrus; e, estrus; m, metestrus; dpc, days *post coitum*; L, labor; dpl, days post-labor. *n* = 4.

**Figure 2:**
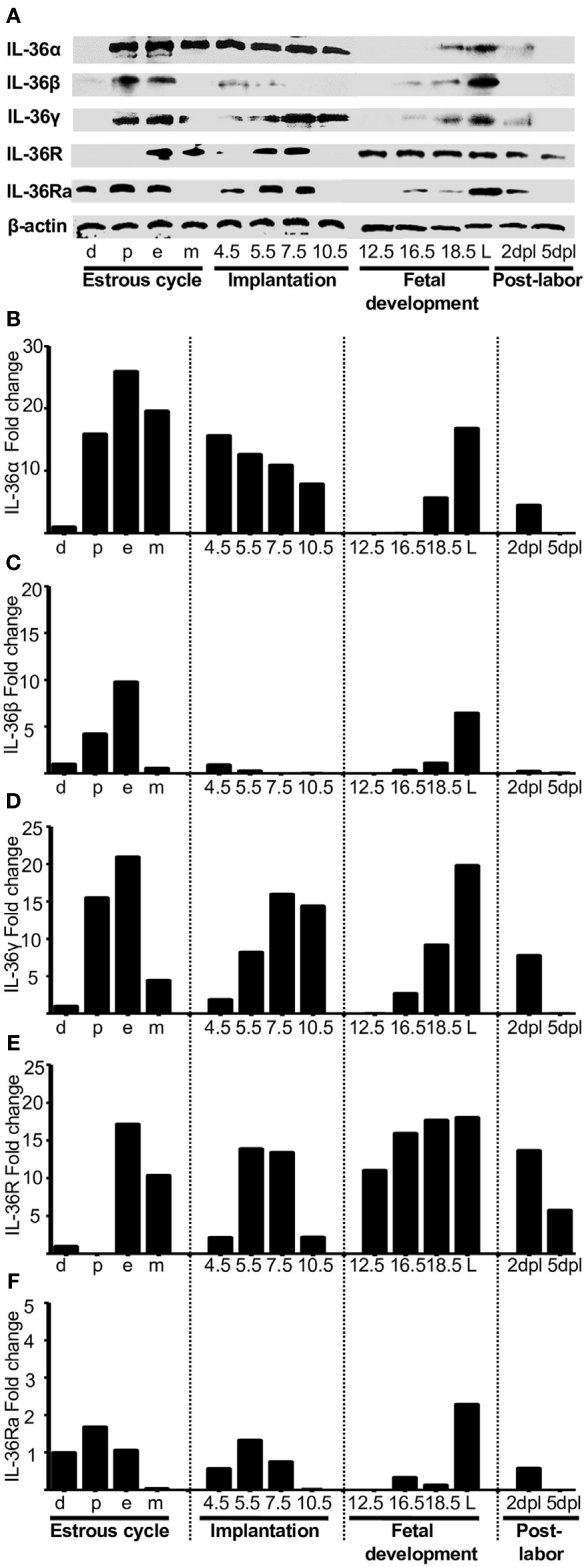
**Protein analyses of IL-36 (α, β, and γ), IL-36R, and IL-36Ra**. Uterus extracts were tested with antibodies against IL-36α, β, γ, IL-36R, and IL-36Ra. Actin was used as a loading control. (**A)** Western blot of representative data from four individuals. (**B–F)** Protein levels are expressed as fold increases normalized to GAPDH and relative to diestrus. d, diestrus; p, proestrus; e, estrus; m, metestrus; dpc, days *post coitum*; L, labor; dpl, days post-labor.

The mRNA expression of IL-36α, β, and γ (Figures 1A–C, respectively) and the corresponding production of proteins (Figures 2A–C, respectively) were similar to those of other pro-inflammatory cytokines in the estrous cycle. That is, expression occurred at diestrus, increased slightly at proestrus, reached maximal expression at estrus, decreased at metestrus, was again lowest at diestrus, and then the cycle started again (Figures 1A–C). It was found that mRNA (Figure 1D) and protein (Figure 2E) levels of IL-36R, which behaved as agonists during the estrous cycle, showed maximal expression at estrus, declined at metestrus, and then was reduced to the baseline level at diestrus. IL-36Ra showed a different expression behavior than the other subfamily members: mRNA (Figure 1E) and protein (Figure 2F) were present mainly at proestrus rather than at estrus; and at estrus and metestrus, expression was even lower than that at proestrus.

### IL-36 Subfamily Members in Embryo Implantation

Embryo implantation is a crucial event for mammals, and inflammatory cytokines are involved. Here, we investigated whether IL-36 subfamily members are involved in implantation.

At the start of implantation, IL-36α expression remained at similar levels at metestrus and decreased at the end of implantation (Figures 1A and 2B). IL-36β expression increased at the beginning of implantation (4.5 dpc) and decreased progressively until 10.5 dpc, and even at 7.5 and 10.5 dpc, the protein could not be detected (Figure 2C). IL-36γ expression was barely detected at the beginning of implantation but had increased by 11-fold at the end of implantation (10.5 dpc) (Figure 1C); IL-36γ protein showed identical behavior (Figure 2D). Expression of IL-36R mRNA remained similar during implantation but increased slightly at 5.5 dpc (Figure 1D). However, expression of IL-36R protein increased at 5.5 and 7.5 dpc during implantation (Figure 2E). Expression of IL-36Ra increased noticeably after metestrus, reaching optimal expression at 5.5 dpc for mRNA and protein (Figures 1E and 2F).

### IL-36 Subfamily Members in Fetal Development

At 12.5 dpc, protein expression of IL-36α, β, and γ was not detectable and increased progressively until labor. Expression of IL-36β mRNA increased considerably, but expression of the protein of IL-36α and γ increased even more (Figures 1E and 2F).

For IL-36R, mRNA expression remained at the same level as during implantation, but the protein increased considerably during fetal development until labor (Figures 1E and 2F). Expression of IL-36Ra increased after 10.5 dpc, reaching optimal mRNA expression at 18.5 dpc, and decreased at labor (Figure 1E).

### Behavior of IL-36 Members after Labor

After a considerable increase in expression at labor, expression of pro-inflammatory cytokines declined after childbirth to hormonal-cycle levels. We also analyzed the behavior of IL-36 subfamily members after labor. Expression of all IL-36 agonists decreased significantly after labor. IL-36R was detected constantly during late pregnancy, even in labor, where production of IL-36 agonists was increased (Figure 1D), but that of their proteins decreased at 5 dpc (Figure 2E). Expression of IL-36Ra was also downregulated after labor (Figures 1E and 2F).

### Immunolocalization of IL-36 in Uterine Tissues

**Figure 3:**
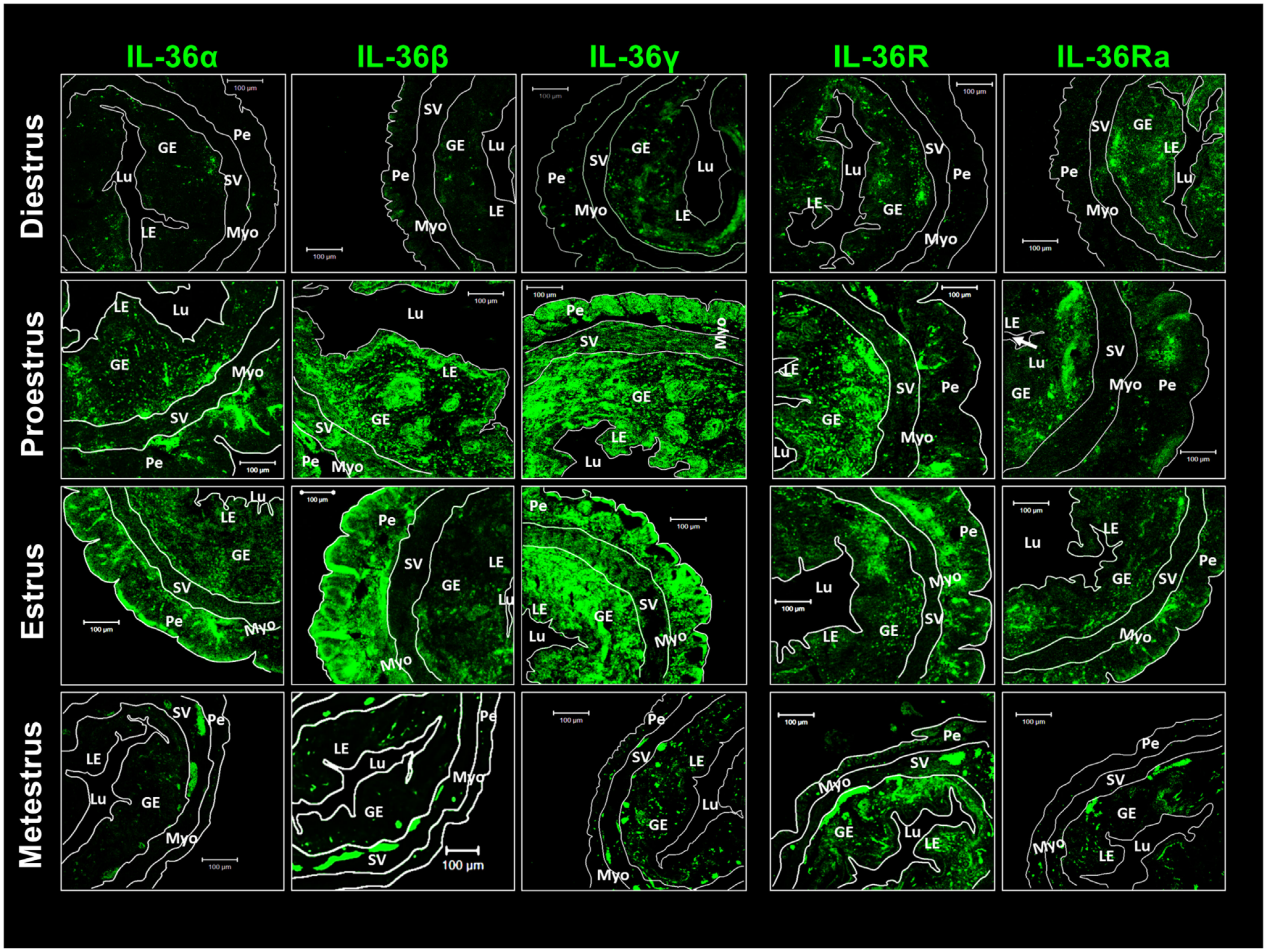
**Pro-inflammatory IL-36 agonists, IL-36R and IL-36Ra are expressed mainly in endometrial and peri-myometrial tissue in the estrous cycle**. ICR females were killed at each stage of the estrous cycle. Tissues slides were treated with goat anti-IL-36 (α, β, γ, R, and Ra) as primary antibody and anti-goat-FITC as secondary antibody. Immunofluorescence images were taken with a LSM 700 confocal microscope (Zeiss) and processed using LSM 5 Image Examiner software (Zeiss). Magnification = × 20. Myo, myometrium; LE, luminal epithelium; GE, glandular epithelium; Lu, lumen; SV, stratum vasculare; Pe, perimetrium.

Immunofluorescence detection of IL-36 subfamily members was undertaken. Diestrus showed a reduced presence of agonists distributed randomly in the luminal epithelium, glandular epithelium, and perimetrium. However, IL-36R and IL-36Ra showed a more concentrated presence in glandular and luminal epithelia (Figure 3). Western blotting revealed the presence of the three agonists to be increased during proestrus and estrus and, in these stages, IL-36α showed a characteristic distribution in the glandular epithelium, perimetrium, myometrium, and stratum vasculare. IL-36β and γ were distributed more homogenously, but spots in the glandular epithelium were not seen. IL-36R and IL-36Ra showed a similar pattern of distribution in diestrus, and they were detected in glandular and luminal epithelia, with increased detection in the perimetrium (Figure 3). At the end of the estrous cycle, during metestrus, detection of agonists and antagonists of IL-36 was diminished drastically compared with that at estrus, whereas IL-36R was maintained in the uterus (Figure 3).

**Figure 4:**
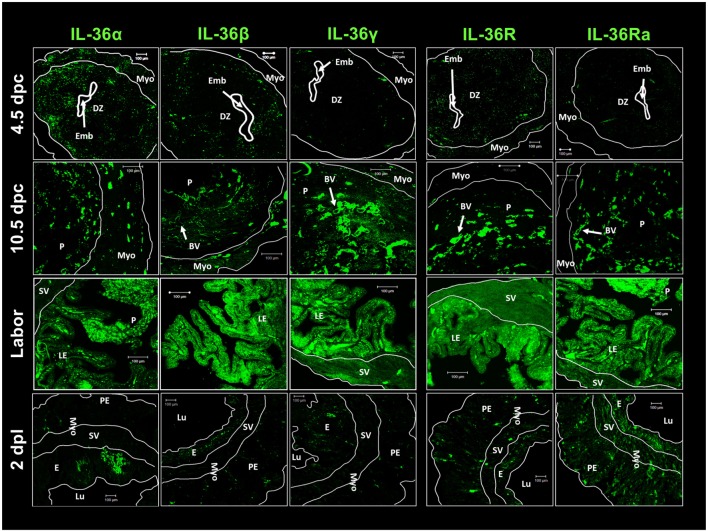
**IL-36 cytokines and IL-36R localization in maternal–fetal tissues during pregnancy**. ICR females were killed at each stage of pregnancy. Tissues slides were treated with goat anti-IL-36 (α, β, γ, R, and Ra) as primary antibody and anti-goat-FITC as secondary antibody. Immunofluorescence images were taken with a LSM 700 confocal microscope (Zeiss) and processed using LSM 5 Image Examiner software (Zeiss). Magnification = × 20 objective. DZ, decidua; Emb, embryo; Myo, myometrium; P, placenta; BV, blood vessels; E, endometrium; Lu, lumen; SV, stratum vasculare; Pe, perimetrium; LE, luminal epithelium; dpc, days *post-coitum*s; dpl, days post-labor.

Expression of the agonists, receptors, and antagonists of IL-36 was localized by immunofluorescence in uterine tissues at early implantation (4.5 dpc), during the final phase of implantation (10.5 dpc), at culmination of pregnancy (labor), and at 2 days after labor (Figure 4). Similar to previous results, expression of IL-36α and IL-36β was higher than IL-36γ expression in the implantation period (4.5 dpc), and this expression was observed in the decidua. IL-36R showed low expression in the decidua and myometrium, as did IL-36Ra (Figure 4).

IL-36 subfamily members at 4.5 and 10.5 dpc showed a characteristic distribution as small spots close to blood vessels. At labor, expression of the five members of the IL-36 subfamily was upregulated (in accordance with western blotting results) and distributed alongside the interface between the luminal epithelium and placenta (Figure 4). Two days after labor, cells from luminal and glandular epithelia showed a large decrease in the production of IL-36 family members, except for moderate expression of IL-36Ra (Figure 4).

### Infection by *Listeria monocytogenes* Induces Severe Overexpression of IL-36 Subfamily Members

We measured the expression of IL-36 subfamily members in a murine model of *L. monocytogenes* infection because infection with this pathogen induces an inflammatory response and can eventually cause fetal loss (11). The infection was established before pregnancy in order to ascertain whether the inflammatory response that occurs normally during implantation in healthy pregnancies would be modified due to *L. monocytogenes*.

**Figure 5:**
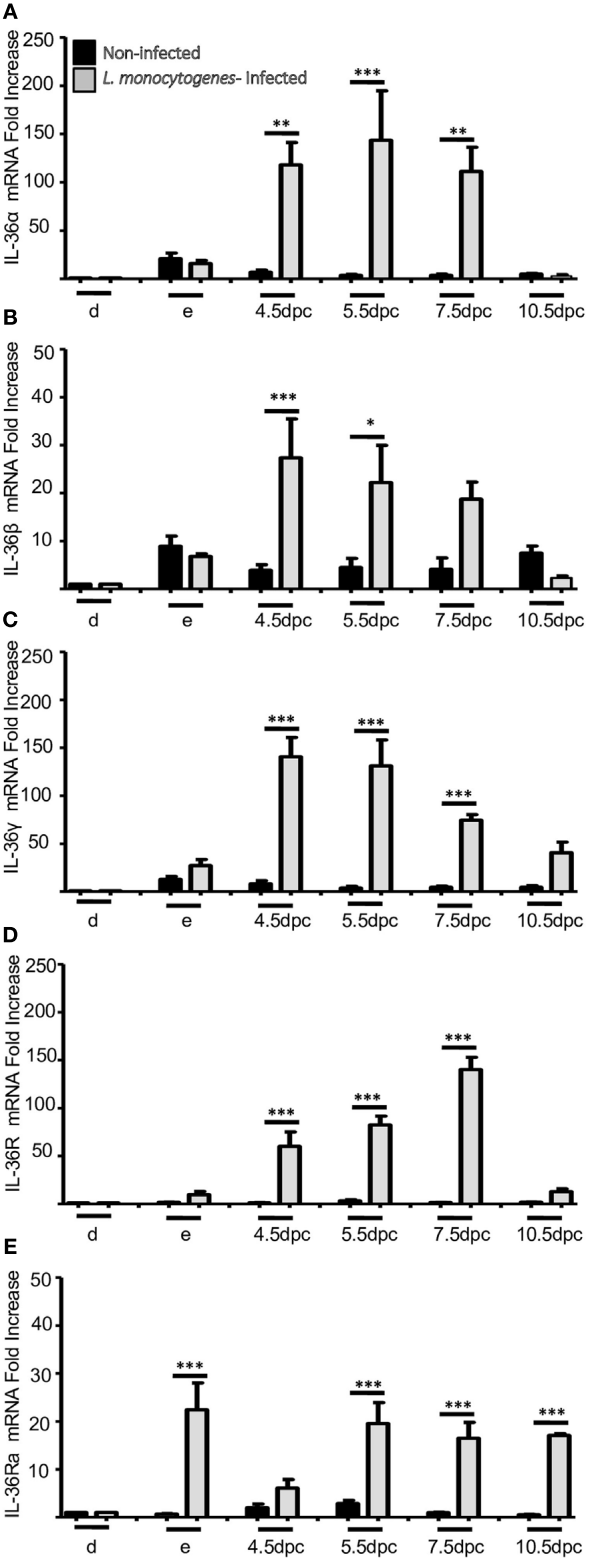
**mRNA expression of IL-36 family members in uterus of infected mice with *L. monocytogenes***. IL-36α **(A)**, β **(B)**, γ **(C)**, IL-36R **(D)**, and IL-36Ra **(E)**. mRNA levels are expressed as fold increases normalized to GAPDH and relative to diestrus of non-infected mice. PCR results are shown as the mean ± SE. *p ≤ 0.05, **p ≤ 0.01, ***p ≤ 0.001 for significant differences between uninfected and infected groups. d, diestrus; e, estrus; dpc, days *post-coitum*. *n* = 4.

**Figure 6:**
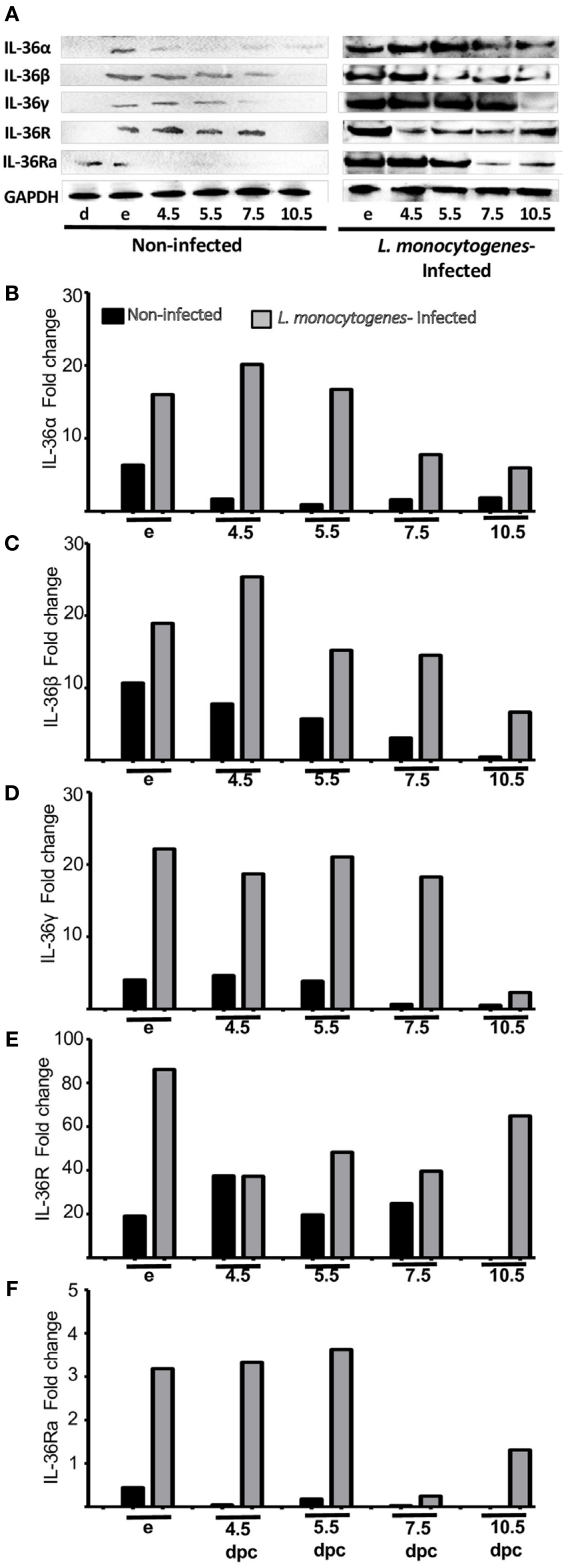
**Protein analyses of IL-36 (α, β, and γ), IL-36R, and IL-36Ra in uterus of mice infected with *L. monocytogenes***. Uterus extracts of non-infected and infected mice were analyzed using antibodies against IL-36α, β, γ, IL-36R, and IL-36Ra. Actin was used as a loading control. **(A)** Western blot of representative data from four individuals. **(B–F)** Protein levels are expressed as fold increases normalized to GAPDH and relative to diestrus of non-infected mice. e, estrus; dpc, days *post coitum*.

Infection by *L. monocytogenes* reduced the number of implanted embryos (data not shown) and induced severe overexpression of IL-36 subfamily members compared with non-infected mice (Figures 5 and 6). Notably, in the uteri of infected mice, the five members of the IL-36 subfamily showed increases in mRNA expression between 25- and 150-fold higher as compared with expression in the implantation sites of non-infected mice at 4.5, 5.5, and 7.5 dpc (Figure 5). Protein analyses revealed a clear increase in expression of IL-36 subfamily members (Figure 6), suggesting that they have a crucial role in the response against *L. monocytogenes*, and that the increased expression could be the cause of miscarriage or infertility (Figure 6).

## Discussion

We found that the expression of IL-36 subfamily members in mice is regulated differently during pro-inflammatory stages in the estrous cycle and in pregnancy, and that they are induced severely by infection during pregnancy. When we evaluated the production of IL-36 subfamily members in mice uteri during the estrous cycle, we found that IL-36 agonists showed a cyclic behavior similar to that observed for other inflammatory cytokines, such as IL-1β, TNFα, and IL-6 (12).

In early pregnancy, implantation is considered to be a fundamental period in which the fertilized egg must interact with the endometrial epithelium that, in turn, must be suitable to receive the product (13). This interaction is regulated strongly by an environment rich in pro-inflammatory cytokines and growth factors that favor blastocyst implantation (14). In early pregnancy, we found that IL-36 subfamily members were produced in different patterns but coincided in their overexpression at estrus where, in the case of egg fertilization, the endometrium is ready for implantation. IL-36α, β, and γ have been shown to induce the cytokine granulocyte-macrophage colony-stimulating factor (GM-CSF) *in vitro* in bone-marrow DCs (15). GM-CSF has been reported to be an important growth factor involved in embryo and placental development in early pregnancy (16).

During the final phase of pregnancy, the fetus has completed its development and is ready for birth. For labor, an influx of immune cells into the myometrium is needed to promote reversal of the inflammatory process. This pro-inflammatory environment promotes contraction of the uterus, expulsion of the fetus, and rejection of the placenta (17).

In healthy pregnancies, we found IL-36α, β, and γ to be highly expressed in labor. In this context, IL-36 has been reported to induce the expression of other pro-inflammatory cytokines, such as IL-1β, IL-6, TNF-α, IL-17, IL-23, IL-12, IL-8, prostaglandin-E2, cyclo-oxygenase-2, and interferon-γ (9, 15, 18). IL-36 also induces expression of chemokines, such as CXCL1, CCL1, CXCL10, CCL11, CCL4, CXCL2, CCL2, CXCL16, CCL7, and CCL20, along with a range of growth factors (GM-CSF, G-CSF, M-CSF, and VEGF) (19), and also metalloproteinases (MMP1, MMP9, MMP10, and MMP19) (20). Expression of most of these molecules is upregulated and has been described during parturition (21).

Finally, we measured the expression of IL-36 subfamily members in mice infected with *L. monocytogenes* before pregnancy. *L. monocytogenes* is a Gram-positive bacterium that can grow and replicate within host cells and that can lead to infertility, spontaneous abortion, premature labor, meningitis, or stillbirth (11). We found that expression of the agonists IL-36α, β, and γ, IL-36R, and that of the antagonist IL-36RA increased by many fold in the uterine tissue of infected pregnant mice compared with non-infected pregnant mice. IL-36 has been associated with a response to bacterial infections. For example, Medina-Contreras and coworkers reported that gut microbiota are involved specifically in IL-36γ expression, which is essential for the resolution of damage induced by dextran sodium sulfate (DSS) (22). Segueni et al. showed that, in a model of systemic infection by *Mycobacterium bovis* attenuated after instillation of Bacillus Calmette–Guérin, IL-36γ expression was increased in the lungs of infected mice (23). Also, it has been shown that IL-36γ has a crucial role in the innate immune response to *Klebsiella pneumoniae* and *Streptococcus pneumoniae* infection, inducing Th-1 and Th-17 immune responses (24).

Non-immune and immune cells, such as keratinocytes, fibroblasts, bronchial epithelial cells, DCs, macrophages, natural killer cells, CD4^+^ lymphocytes, and neutrophils, are important sources of IL-36 cytokines if they come into contact with certain pathogen-associated molecular patterns, and could be important for the immune response against *L. monocytogenes* (25). Neutrophil-produced proteases, such as cathepsin G, elastase, and proteinase-3, are responsible for cleavage of IL-36α, β, γ and IL-36Ra to obtain active agonist or antagonist fractions (26, 27). Neutrophils are one of the first cell types in the innate immune system to respond, so it seems that processed IL-36 agonists have a critical role in the initial response to *L. monocytogenes* infection or tissue damage at the maternal–fetal interface. However, *L. monocytogenes* uses phagocytic cells for dissemination; we do not know the main cell type responsible for IL-36 production and whether the overproduction of IL-36 induced by *L. monocytogenes* contributes to the elimination of this pathogen or to its dissemination toward fetal tissues.

In human gestational tissues, IL-36 expression has not been studied thoroughly. Only Southcombe et al. have reported no differential expression of IL-36α, β, and γ in the placentas of women with healthy pregnancies compared with women with preeclampsia (28). However, they found lower production of IL-36Ra in the sera and placenta of women with preeclampsia compared with normal pregnancies (28). Our results suggest that the IL-36 system may have important roles for (i) progression of the estrous cycle; (ii) maintenance of the inflammatory state during embryo implantation; and (iii) successful labor.

## Conclusion

We show, for the first time, differential IL-36 (α, β, γ, R, and Ra) mRNA expression and protein production profile in the reproductive tract during the estrous cycle, pregnancy, and labor in healthy pregnancies of mice, and during embryo implantation in infected pregnant mice. IL-36 agonists showed a pattern of expression and production similar to that seen for other pro-inflammatory cytokines reported previously.

## Author Contributions

All authors listed, have made substantial, direct and intellectual contribution to the work, and approved it for publication.

## Conflict of Interest Statement

All authors declare no conflict of interests, including any financial, personal, or other relationships with other people or organizations that could influence (or be perceived to influence) the results in an inappropriate manner.
